# Medical Management of Obesity: A Comprehensive Review of Food and Drug Administration (FDA)-Approved and Investigational Therapies

**DOI:** 10.7759/cureus.96739

**Published:** 2025-11-13

**Authors:** Syed S Raza, Zarshal Zakir, Ahmad Hashmat, Saira K Awan, Giustino Varrassi

**Affiliations:** 1 Family Medicine, The Wright Center for Graduate Medical Education, Scranton, USA; 2 Internal Medicine, Commonwealth Health Regional Hospital of Scranton, Scranton, USA; 3 Medicine, CHI St. Alexius Health, Bismarck, USA; 4 Nephrology, Richmond University Medical Center, New York, USA; 5 Neurology, Advanced Neurology P.C., Brooklyn, USA; 6 Medicine, Kabir Medical College, Peshawar, PAK; 7 Pain Medicine, Fondazione Paolo Procacci, Rome, ITA

**Keywords:** bmi management, glp-1 agonist, glp-1-gip co-agonist, high body mass index, obesity, weight loss

## Abstract

The global rise in obesity has accelerated both clinical and pharmaceutical innovation in antiobesity pharmacotherapy. This narrative review synthesizes current evidence on Food and Drug Administration-approved medications and emerging investigational agents that are shaping clinical practice. We summarize mechanisms of action, pivotal efficacy data, safety profiles, indications, prescribing guidance, and key uncertainties. Approved long-term agents, orlistat, phentermine/topiramate, naltrexone/bupropion, liraglutide, semaglutide, and tirzepatide, differ in mechanism, weight-loss magnitude, and safety considerations. Semaglutide and tirzepatide have redefined expectations for pharmacological weight loss, while next-generation drugs, such as oral glucagon-like peptide 1 receptor agonists (e.g., orforglipron) and multireceptor agonists (e.g., retatrutide), show even greater efficacy in early studies. Common safety concerns include gastrointestinal effects, gallbladder events, pancreatitis risk, thyroid C-cell tumor warnings, teratogenicity, and cost barriers. Appropriate patient selection depends on body mass index, comorbidities, contraindications, and treatment goals, with close monitoring throughout therapy. Long-term data on cardiovascular outcomes and posttreatment weight durability are emerging. Future research should prioritize direct comparative trials, real-world effectiveness, long-term safety, and strategies to improve access and adherence. This review offers clinicians a concise, evidence-based guide for obesity pharmacotherapy and outlines key research priorities as the treatment landscape rapidly evolves.

## Introduction and background

Obesity (body mass index (BMI) ≥30 kg/m²) increases the risk of diabetes, heart disease, sleep apnea, some cancers, and poor quality of life. Medicines can help when lifestyle change is not enough. Current long-term options include orlistat, phentermine/topiramate, naltrexone/bupropion, liraglutide, semaglutide, and tirzepatide [1-9]. Each has a different mechanism, weight loss profile, dose schedule, and risk pattern. In the last decade, incretin-based drugs changed what clinicians can expect from pharmacotherapy, moving typical weight reductions from single digits to double digits in many patients [5,6,10]. Semaglutide and tirzepatide have set new expectations for average weight loss in randomized trials.

Obesity involves abnormal appetite signaling and energy balance. Approved drugs target three broad paths. The first pathway reduces fat absorption (orlistat). The second pathway reduces appetite centrally (typically seen with phentermine/topiramate and naltrexone/bupropion). The third pathway uses incretin biology to reduce appetite, slow gastric emptying, and improve metabolic control (liraglutide, semaglutide, and tirzepatide) [1-6,9,11].

The STEP 1 clinical trial found that semaglutide 2.4 mg once weekly resulted in approximately 15% mean weight loss at 68 weeks, compared to 2%-3% with placebo [5]. In SURMOUNT-1, tirzepatide led to a 15%-21% loss at 72 weeks, depending on dose titration [6]. Early trials of next-generation agents, oral small-molecule glucagon-like peptide 1 (GLP-1) agonists such as orforglipron and triple agonists such as retatrutide, show even greater or comparable efficacy and new routes of administration [7,8].

Given the emergence of newer weight loss management medications, safety and monitoring remain central. Common side effects encountered include gastrointestinal (GI) symptoms. GLP-1-based drugs carry warnings for gallbladder events and rare pancreatitis. They also carry a class boxed warning about medullary thyroid carcinoma based on rodent data; patients with a personal or family history of medullary thyroid cancer (MTC) or multiple endocrine neoplasia 2 (MEN2) should not receive these agents [9]. Other class-specific issues include teratogenicity with topiramate in the phentermine/topiramate combination. Seizure and blood pressure (BP) concerns have been noted with naltrexone/bupropion combination [2,4].

Choosing the right drug means matching indications (BMI thresholds and comorbidities), goals, contraindications (including pregnancy), access, and patient preferences [1,2,9,11]. Emerging data now include cardiovascular outcomes for semaglutide 2.4 mg in high-risk patients without diabetes (SELECT) [10].

This review explains how current Food and Drug Administration (FDA)-approved drugs work, how well they perform in pivotal trials, and how to use them safely and practically. It also highlights promising agents in development.

## Review

Methodology

We conducted a narrative review to gather and summarize FDA-approved and investigational therapies in the medical management of obesity. We structured our work around the Scale for the Assessment of Narrative Review Articles criteria to keep the process clear, rigorous, and relevant [12].

Search Strategy

We searched PubMed, MEDLINE, EMBASE, Google Scholar, Scopus, and the Cochrane Library for studies published from 1990 to 2025. We combined terms such as “obesity pharmacotherapy”, “GLP-1”, “semaglutide”, “tirzepatide”, “retatrutide”, and “orforglipron”. We prioritized pivotal randomized trials, FDA labeling, and influential guidelines [1-11]. We cite representative sources for the main claims.

Pathophysiologic rationale for pharmacotherapy

Obesity reflects a dysregulated energy balance, characterized by complex interactions among central appetite circuits, incretin signaling, gut hormones, adipokines, and peripheral metabolic tissues. Modern pharmacotherapy targets three pathways. The first pathway reduces fat absorption (orlistat). The second pathway reduces appetite centrally (typically seen with phentermine/topiramate and naltrexone/bupropion). The third pathway uses incretin biology to reduce appetite, slow gastric emptying, and improve metabolic control (liraglutide, semaglutide, and tirzepatide) [1-6,9,11]. The last class has produced the largest mean weight loss in controlled trials to date.

FDA-approved agents: overview and comparative summary

Several agents are FDA-approved for chronic weight management; selection depends on BMI and comorbidities, prior response to lifestyle interventions, contraindications, and patient preference.

Orlistat

It is a lipase inhibitor that blocks nearly 30% dietary fat absorption. Typical weight loss over placebo is modest (3%-5%). The common side effects are oily stools, urgency, and reduced absorption of fat-soluble vitamins. Patients taking this drug should be given a daily multivitamin at a separate time. Orlistat is contraindicated in chronic malabsorption [1,13,14].

Phentermine/Topiramate

This drug combination consists of a sympathomimetic (phentermine) and topiramate (reduces weight mainly by lowering food intake and cravings by inhibiting carbonic anhydrase, which can cause dysgeusia and mild metabolic acidosis, both linked to reduced appetite). The mean weight loss observed is 8%-10% at 56 weeks, compared to 1% with the placebo [2]. In female patients, it is advisable to use contraception and monthly pregnancy tests due to the risk of teratogenicity [2].

Naltrexone/Bupropion

This combination modulates reward pathways and appetite. Phase 3 COR trials show a modest additional loss compared to placebo, especially with behavioral support [3,4]. Contraindications include seizure disorder, uncontrolled hypertension, chronic opioid use, and eating disorders. Patients are advised to monitor BP and mood [4].

Liraglutide

This is an injectable GLP-1 agonist taken daily as a subcutaneous injection. The SCALE (Satiety and Clinical Adiposity - Liraglutide Evidence in Nondiabetic and Diabetic Individuals Obesity and Prediabetes) trial found a mean weight loss of 8% vs. 2-3% with placebo at 56 weeks [5]. Adverse effects include GI symptoms, gallbladder events, rare pancreatitis, and boxed thyroid C-cell tumor warning based on rodent data, and avoid in MTC/MEN2 [9,11].

Semaglutide

This is an injectable GLP-1 agonist medication taken as subcutaneous injections. The STEP 1 trial found a mean loss of 15% at 68 weeks, compared to 2%-3% with placebo [5]. The SELECT trial enrolled 17,600 participants and showed fewer major adverse cardiovascular events in patients with obesity and established cardiovascular disease without diabetes [10]. This drug has a safety profile similar to liraglutide and a boxed warning as above [5,9,10].

Tirzepatide

This is also an injectable drug taken weekly, but it is a dual gastric inhibitory polypeptide (GIP)/GLP-1 agonist. The SURMOUNT-1 trial showed a 15%-21% mean weight loss at 72 weeks of use [6]. GI effects are common; class warnings are similar to GLP-1 agonists [6,9].

**Figure 1:**
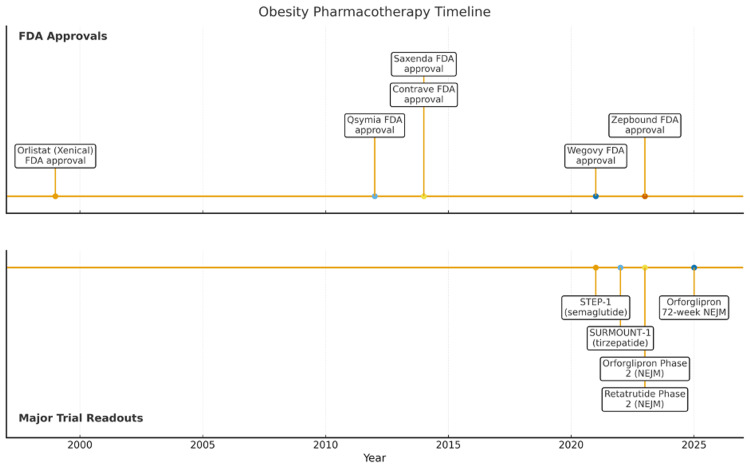
Timeline figure (approvals and major readouts 1999-2025) The figure outlines a timeline of the major obesity management trials from 1999 to 2025 FDA: Food and Drug Administration; NEJM: New England Journal of Medicine

In this review, we created a simple horizontal timeline of key FDA approvals and landmark trials between 1999 and 2025 (Table 1 and Figure 1).

**Table 1:** Obesity pharmacotherapy: approvals and major trial readouts (1999-2025) FDA: Food and Drug Administration; GLP-1: glucagon-like peptide 1

Year	Type	Pharmacological agent	Evidence
1999	FDA approval	Orlistat	[1,13,14]
2012	FDA approval	Phentermine/topiramate	[2]
2014	FDA approval	Naltrexone/bupropion	[3]
2014	FDA approval	Liraglutide	[3,4,11]
2021	FDA approval + trial	Semaglutide	[5,9-11]
2023	FDA approval	Tirzepatide	[6]
2023	Trial	Retatrutide (triple agonist)	[8]
2023	Trial	Orforglipron (oral GLP-1)	[7]

Quick comparison

A comparison of approved antiobesity medications, their typical weight loss, dosing, and key cautions from the different trials is given in Table 2.

**Table 2:** Summary of antiobesity pharmacotherapies: efficacy, dosing, and key considerations Summary of approved antiobesity medications and expected mean percent weight change from pivotal trials. Effects are approximate and vary by patient and adherence TID: ter in die (three times a day); GI: gastrointestinal; REMS: risk evaluation and mitigation strategy; HTN: hypertension; SC: subcutaneous (injection under the skin); AEs: adverse events/effects; CV: cardiovascular

Medication	Typical weight loss and duration	Dosing	Key cautions/notes	Evidence
Orlistat	Modest additional loss vs. lifestyle alone	Oral, TID with fat containing meals	Strong GI effects; fat-soluble vitamin supplementation needed	[1,13,14]
Phentermine/topiramate	8%-10% at 1 year; sustained at 2 years	Oral, daily	Teratogenic risk, avoid in pregnancy; REMS	[2]
Naltrexone/bupropion	Modest-moderate loss	Oral, daily	Avoid in seizures, uncontrolled HTN, or on opioids	[3,4]
Liraglutide 3.0 mg	8% at 56 weeks	SC injection, daily	GI AEs common; titration needed	[5]
Semaglutide 2.4 mg	15% at 68 weeks; CV benefit shown in SELECT trial	SC injection, weekly	GI AEs; dose escalation	[5,10]
Tirzepatide	15%-21% at 72 weeks	SC injection, weekly	GI AEs; dose escalation	[6]

Investigational and emerging therapies

Rapid innovation in obesity pharmacology has led to the emergence of newer, better, safer weight loss medications with improved efficacy, alternate routes (oral), and multireceptor agonists [6]. Orforglipron is an investigational drug. It is an oral, small-molecule GLP-1 agonist. Phase 2 trial showed meaningful weight loss, while phase 3 programs are ongoing, and early readouts are positive [7]. Similarly, retatrutide is another investigational triple agonist (GLP-1/GIP/glucagon), designed to maximize weight loss via multiaxis metabolic modulation. Phase 2 data show very large mean losses (20%-24% at 48 weeks at higher doses) with broad metabolic gains. Phase 3 is in progress [8].

Practical prescribing guidance

Most antiobesity labels qualify adults with BMI ≥30 or ≥27 kg/m² plus a weight-related comorbidity (diabetes, hypertension, dyslipidemia, and obstructive sleep apnea) [1,11]. While prescribing clinicians need to set realistic but impactful goals, a 5%-10% weight loss yields clinically meaningful benefits. However, many patients on semaglutide or tirzepatide can target ≥15% if treatment is tolerated and accessible [5,6]. Drug selection should be individualized to comorbidities, route (oral vs. injection), contraindications, patient preference, and insurance coverage. In women of childbearing age where pregnancy is possible, avoid teratogens and ensure effective contraception, especially with phentermine/topiramate, due to their risk of teratogenicity [2,4,9,11]. Therapy should be initiated at the lowest effective dose and titrated gradually to mitigate GI adverse effects. Weight loss, glycemia, mood, BP, and other adverse events should be monitored [5,6,11].

Access, cost, and equity

It is noteworthy that cost and insurance rules limit the use of weight loss medications. Oral agents and future generics may also be helpful. System solutions, coverage changes, and value-based contracts are needed to translate trial efficacy into population benefit [11]. Antiobesity medications work well, but the real question remains around their uneven access. Medication coverage varies by plan rules, payer, and indication. Medicare still cannot cover drugs for obesity alone. However, since the FDA added a cardiovascular risk reduction indication for semaglutide, many part D plans began covering it for cardiovascular-related indications, but not necessarily for weight loss. Coverage still remains plan-specific and inconsistent [15-17].

Another barrier to access is the out-of-pocket cost. Even with deductibles, insurance, and prior authorization (PA), steps can delay or block medication fills. However, for self-pay patients, manufacturers now offer direct programs that set transparent cash prices on a monthly or membership basis that bypass insurance but do not count toward deductibles. These costs lower the ceiling but still exceed what many needy patients can afford [18,19].

The data show a large discontinuity and fill-fail rates that track with coverage and cost. In a 2025 cohort, nearly 40% of GLP-1 prescriptions were never filled. Hispanic and Black patients were less likely to fill than White patients. Another study found that most patients were not able to continue GLP-1s within a year of initiation, with higher discontinuation rates among those without diabetes. These patterns risk widening disparities in obesity outcomes [19,20].

Furthermore, equity concerns go beyond price. Different formularies may require diabetes co-diagnoses, higher BMI thresholds, or step therapy that privilege some groups and exclude others. A 2024 study highlights PA burdens and restrictive criteria across major commercial plans. These policies can produce uneven access by geography, income, and race/ethnicity [16,21].

The bottom line is that administrative hurdles, cost, and coverage rules now shape who benefits from these medicines as much as clinical eligibility does. Broadening indication-aligned coverage, reducing PA friction, and monitoring filling gaps are immediate steps to turn trial efficacy into population-level benefit [16].

Future directions and research priorities

The current medical literature lacks evidence on Head-to-Head trials among semaglutide, tirzepatide, triple agonists, and oral GLP-1s [5-8,10]. Long-term safety, durability after discontinuation, and outcomes beyond weight (CV events, cancer, and mortality) need to be studied in detail for the medication to be used in the general population [5-8,10,11].

Limitations

This narrative synthesis is not a systematic review and therefore may not capture every trial or dataset. Evidence continues to evolve rapidly (notably in 2025), and readers should consult the latest FDA labeling and pivotal trial publications when making clinical decisions.

## Conclusions

Modern antiobesity drugs have changed clinical care by making double-digit weight loss achievable for many patients. GLP-1-based and dual agonist therapies now lower weight and, in some cases, improve cardiometabolic risk, which moves treatment beyond short-term weight control. Access and cost remain major barriers, and they shape who actually receives these therapies and who can stay on them. Emerging agents, including oral GLP-1 receptor agonists and triple agonists, may offer greater effect and easier delivery. Ongoing work needs to focus on durability after stopping therapy, long-term safety, head-to-head comparisons, and more equitable coverage so these benefits reach routine care.

## References

[1] Pi-Sunyer X, Astrup A, Fujioka K (2015). A randomized, controlled trial of 3.0 mg of liraglutide in weight management. N Engl J Med.

[2] Gadde KM, Allison DB, Ryan DH, Peterson CA, Troupin B, Schwiers ML, Day WW (2011). Effects of low-dose, controlled-release, phentermine plus topiramate combination on weight and associated comorbidities in overweight and obese adults (CONQUER): a randomised, placebo-controlled, phase 3 trial. Lancet.

[3] Apovian CM, Aronne L, Rubino D (2013). A randomized, phase 3 trial of naltrexone SR/bupropion SR on weight and obesity-related risk factors (COR-II). Obesity (Silver Spring).

[4] Rubino D, Schon S (2025). Treating obesity to optimize women's health outcomes. Menopause.

[5] Wilding JP, Batterham RL, Calanna S (2021). Once-weekly semaglutide in adults with overweight or obesity. N Engl J Med.

[6] Jastreboff AM, Aronne LJ, Ahmad NN (2022). Tirzepatide once weekly for the treatment of obesity. N Engl J Med.

[7] Wharton S, Blevins T, Connery L (2023). Daily oral GLP-1 receptor agonist orforglipron for adults with obesity. N Engl J Med.

[8] Jastreboff AM, Kaplan LM, Frías JP (2023). Triple-hormone-receptor agonist retatrutide for obesity - a phase 2 trial. N Engl J Med.

[9] Spitery A, Elder MJ, Farhat N, Mohammad I, Lobkovich A (2024). Legal, safety, and practical considerations of compounded injectable semaglutide. Am Coll Clin Pharm.

[10] Lingvay I, Brown-Frandsen K, Colhoun HM (2023). Semaglutide for cardiovascular event reduction in people with overweight or obesity: SELECT study baseline characteristics. Obesity (Silver Spring).

[11] Grunvald E, Shah R, Hernaez R (2022). AGA clinical practice guideline on pharmacological interventions for adults with obesity. Gastroenterology.

[12] Baethge C, Goldbeck-Wood S, Mertens S (2019). SANRA-a scale for the quality assessment of narrative review articles. Res Integr Peer Rev.

[13] Hendricks EJ (2017). Off-label drugs for weight management. Diabetes Metab Syndr Obes.

[14] Gasmi A, Mujawdiya PK, Nehaoua A (2023). Pharmacological treatments and natural biocompounds in weight management. Pharmaceuticals (Basel).

[15] Baig K, Dusetzina SB, Kim DD, Leech AA (2023). Medicare part D coverage of antiobesity medications - challenges and uncertainty ahead. N Engl J Med.

[16] Liu X, Lu CA, Shih YT, Jiang C (2025). Coverage and prior authorization policies for semaglutide and tirzepatide in Medicare part D plans. JAMA Netw Open.

[17] Hwang JH, Laiteerapong N, Huang ES, Mozaffarian D, Fendrick AM, Kim DD (2025). Fiscal impact of expanded Medicare coverage for GLP-1 receptor agonists to treat obesity. JAMA Health Forum.

[18] Hernandez I, Sullivan SD (2024). Net prices of new antiobesity medications. Obesity (Silver Spring).

[19] Sarpatwari A, Soto MJ, Ganguli I, Sloan CE, Goss F, Sinaiko AD (2025). Glucagon-like peptide-1 receptor agonist order fills and out-of-pocket costs by race, ethnicity, and indication. JAMA Health Forum.

[20] Rodriguez PJ, Zhang V, Gratzl S (2025). Discontinuation and reinitiation of dual-labeled GLP-1 receptor agonists among US adults with overweight or obesity. JAMA Netw Open.

[21] Liu BY, Rome BN (2024). State coverage and reimbursement of antiobesity medications in Medicaid. JAMA.

